# Social Justice in Post‐Conflict Societies: Lessons From Northern Ireland

**DOI:** 10.1111/1468-4446.13207

**Published:** 2025-03-22

**Authors:** Ruth McAreavey, Katharine A. M. Wright, Rebecca Donaldson

**Affiliations:** ^1^ School of Geography, Politics and Sociology Newcastle University Newcastle‐upon‐Tyne UK

**Keywords:** Brexit, gender, (mis)Recognition, Northern Ireland, social justice, sovereigntism, women

## Abstract

This article explores gender and social justice in post‐conflict societies, using Northern Ireland as a case study. It focuses specifically on the socio‐economic impact of the UK's withdrawal from the EU (Brexit) on women in Northern Ireland using a social justice framework, drawing on recognition, redistribution and representation as conceptualised by Nancy Fraser. It uses qualitative research conducted between 2022 and 2023 comprising focus groups, an expert seminar and semi‐structured interviews sensitive to an intersectional understanding of women. While centred on Brexit, the findings have broader implications for understanding how post‐conflict governance, sovereignty, and international obligations intersect with gendered inequalities. We argue that Brexit demonstrates a profound neglect of Northern Ireland's unique position, politically and geographically, particularly the UK's obligations under the Belfast (Good Friday) Agreement, and underscores the marginalisation and exclusion of women's voices in post‐conflict governance. We find that the impact of Brexit on women in Northern Ireland is distinct and disproportionate from other parts of the UK for several reasons, including that it is a post‐conflict society; there exists specific patterns of violence against women; and there is a prior reliance by the third sector on EU funding. The article thus contributes to a deeper understanding of the systemic barriers that inhibit participatory equality and outlines pathways for achieving social justice in Northern Ireland.

## 1 Introduction

The history and complexity of Irish‐British relations has persisted for many decades including one‐directional invisibility of Ireland by England (Hickman and Ryan 2020). Recent events relating to the Brexit negotiations, and the eventual agreement of the Windsor Framework, suggest that the complexity of relations prevails. This is due in part to over‐representation of public schooled and Oxford‐educated individuals in politics (Bagehot 2018, cited in Hickman and Ryan 2020); politicians who have perhaps conveniently (Gilligan 2021), failed to grasp the complexity of the issue. Part of the Brexit discourse was about ‘taking back control’ with the aim to ‘reclaim the UK's sovereignty’ (UK Government 2018, 4, see also Calhoun 2016). Research shows how Brexit has had profound and uneven implications across different parts of society, including on family mobilities and on personal relationships (see for instance Zambelli et al. 2024). It has also been shown to have been a highly gendered process, risking women's rights and gender equality in the UK (MacLeavy 2018; Guerrina and Masselot 2018). This has been manifested through the marginalisation of women and ‘women's issues’ across the referendum campaigns, media coverage, academia and in the resulting negotiations from both a UK and EU perspective. Rather, ‘men's concerns’ and masculinised politics including security, the market, family protection became prioritised (Achilleos‐Sarll and Martill 2019). The nature of the Leave and Remain campaigns were also highly adversarial in nature (Usherwood and Wright 2017). Concerns were raised about a clear xenophobic and anti‐immigration line emerging from the ‘leave’ campaigns (Golec De Zavala et al. 2017), thus further disengaging women from Brexit (Guerrina et al. 2018, see also Hozić and True 2017).

An established body of scholarship considers the gendered impact of Brexit in the UK, relating to gender equality legislation (Guerrina and Masselot 2018), worker's rights (Fagan and Rubery 2018) and mobilities (Kilkey et al. 2020). More broadly, research has focussed on how the gendered silences in academic, media and policy work on Brexit have contributed to the siloing of such research (Guerrina and Murphy 2016; Guerrina et al. 2018; Galpin and Vernon 2023). At the regional level within the UK, work has been done on the impact of Brexit on the equalities agenda in Wales (Minto and Parken 2021), and in Scotland, where the impact of Westminster increasingly bypassing subnational governments as a result of the Brexit process resulted in ‘stifling women friendly policy agendas at the devolved level’ (Sanders and Flavell 2023). We build on this literature to contribute to an understanding of the gendered impact of Brexit in Northern Ireland using the framework of social justice (Fraser 2001, 2005) and against a backdrop of ‘sovereigntism^1^’ (Benhabib 2016). This article thus contributes to a deeper understanding of the role and impact of gender in shaping social and economic outcomes in post‐conflict societies. In so doing we pick up on important themes identified in previous literature including the way in which the Irish have been made invisible due to their ‘whiteness and presumed cultural similarity’ (Hickman and Ryan 2020, 99); and the convenience of considering Northern Ireland as a ‘place apart’ (Gilligan 2017, 5).

In this article, we adopt an intersectional approach to examine the impacts of Brexit on women in Northern Ireland, focussing on both their current experiences and future perceptions. This approach recognises the diversity of women's experiences and considers how multiple intersecting identities shape them, paying particular attention to the experiences of women in rural areas, minority ethnic women, LGBTQI + women and disabled women. This means that it is not possible to say ‘there's a race problem here, a gender problem here, and a class or LGBTQ problem there. Many times that framework erases what happens to people who are subject to all of these things’ (Crenshaw 2017).

We understand Brexit as an ongoing process of the UK asserting its sovereignty by exiting the EU. It followed from the announcement of a referendum in 2016 on the UK's membership of the EU by Prime Minister David Cameron in February 2016 and continued after the formal withdrawal agreement of 2019. Brexit is far from done, and as we demonstrate, this is felt acutely in Northern Ireland. Drawing on an intersectional approach, we ask: what impact has Brexit had on social justice for women in Northern Ireland? We situate our study within a backdrop of sovereigntism as elaborated by Benhabib (2016) where she argues that certain states have reverted to a territorial or Westphalian understanding of sovereigntism which is somehow in tension with international obligations. For instance, in the US the sovereigntism of the Supreme Court has long been evident. Meanwhile in Europe we have seen a growing resistance to the European Court of Human Rights with questions being asked about the relevance of decisions being taken in a Strasbourg court (Benhabib 2016). Through this position of sovereigntism, states assume that sovereignty and transnational law are antagonistic whereas as Benhabib argues, the latter can enhance the former and ought to be engaged in ‘a reflexive and iterative hermeneutic’ (2016, 112).

The article is structured as follows. First, we provide important context on Northern Ireland, the peace process and Brexit. We then elaborate on our understanding of Nancy Fraser's conceptualisation of social justice. Next, we examine representation, arguing that misrepresentation has been used to prioritise a gender‐neutral conceptualisation of trade and related issues at the expense of women's interests. We build on this by then considering women's voices within democratic iterations. Following that, we turn to consider the redistribution of resources by examining funding for the third sector, specifically Section 75 groups, and the resultant challenges Brexit has entailed in this area. Next, we analyse equalities legislation and the protection of gender equality in Northern Ireland through a focus on recognition. Finally, we conclude by arguing that Brexit has negatively impacted on social justice outcomes in Northern Ireland due to the UK's failure to understand and account for Northern Ireland's unique socio‐political context. Instead, the UK prioritized a sovereignty‐focused approach, which disregarded critical nuances and international obligations. This highlights the urgent need for robust national and international engagement to uphold human rights, promote equitable resource distribution, and foster inclusive participation for all citizens.

### 1.1 Northern Ireland: The Peace Process and Brexit

Northern Ireland has a unique position within the United Kingdom having experienced decades of civil unrest during much of the latter half of the 20th century. Peace negotiations were brokered by international politicians, especially from the US under Bill Clinton's administration. They notably involved female activists who quickly became politicians under the guise of the Women's Coalition so that they could have a seat at the negotiating table as well as Westminster's Secretary of State to Northern Ireland, Mo Mowlam. The involvement of women has been credited with making the peace settlement (the Belfast/Good Friday Agreement) more relevant to people's lives due to various measures including requiring integrated education, promoting mixed houses and involving youth and victims of violence in reconciliation (Bachelet 2015). The UK Government highlights women's inclusion in the peace process as a means to showcase its engagement with the UN Women, Peace and Security (WPS) agenda. However, this is contradicted by the absence of WPS considerations during the Brexit negotiations, despite Brexit's significant gendered implications for peacebuilding in Northern Ireland (Wright et al. 2024a). As research has shown, even when women are included in peace processes, the gendered structure of these processes, rooted in masculinised norms, often limits their transformative potential (Aggestam and Towns 2018). Therefore, inclusion alone is insufficient to ensure outcomes that address the needs of women and other minoritised groups equitably, leaving them at a disadvantage, including during the peace process in Northern Ireland (McAuliff 2022). Our findings support this and highlight the lack of active consultation with women during the Brexit negotiations and the disproportionate impact of Brexit on women. This underscores the enduring presence of entrenched masculine power structures in both the UK and Northern Ireland, challenging narratives that position Northern Ireland as an exemplar of the WPS agenda given that the peace process has not been transformative of gender relations.

Many other aspects of the resulting Belfast (Good Friday) Agreement (GFA) were both remarkable and innovative, and supportive of women's rights. Notably, multiple commitments were made to uphold equality and human rights including within public administration via Section 75 of the Northern Ireland Act (O'Connell et al. 2024). This includes the need to promote equality of opportunity across nine different categories. In addition, the GFA was made between two sovereign nations, Ireland and the UK, which facilitated the creation of transnational cross‐border institutions. This arrangement was made possible by the shared EU membership of both countries. A fundamental assumption underpinning the GFA was, therefore, that the UK would remain a member of the EU.

While Brexit may be perceived as being ‘done’ in England, Scotland and Wales, it continues to define politics in Northern Ireland as demonstrated by the 2024 UK General Election which was dominated by Brexit politics (The Guardian 2024). Protocol Article 2 (UK Government 2020), subsequently revised through Article two of the Windsor Framework (hereafter referred to as the Windsor Framework) sets out the UK Government's commitments on equality and human rights post Brexit. The Windsor Framework states that, as a result of Brexit, there will be no diminution of rights, safeguards or equality of opportunity as set out in Chapter Six of the GFA. We note that Brexit entwines with other crises impacting on Northern Ireland making its impact sometimes difficult to discern from issues which are solely related to Brexit. This includes the cost‐of‐living crisis; war in Ukraine; Covid‐19 pandemic; healthcare crisis; violence against women; universal credit and longer‐term impacts of austerity.

### 1.2 Social Justice and (Mis)Recognition of Women in Northern Ireland

In arguing for a critical theory of justice, Nancy Fraser refers to the recognition of difference along boundaries of nationality, ethnicity, race,^2^ sexuality and gender. A socially just approach seeks to impede institutional norms that belittle some categories of people or destructive behaviours, attitudes and claims (Fraser 2005). Fraser's framework has been effectively used to examine seasonal worker schemes (McAreavey 2019) and to understand migration and individuals' interactions with social structures (Hölscher 2014). These studies show how many obstacles exist to achieving social justice, including economic inequities; unequal treatment of different social groups; and asymmetric power relations that privilege certain groups while disadvantaging others.

At its simplest, justice is about parity of participation (Fraser 2005). Originally Fraser argued that justice can only be achieved by recognition and redistribution, this forming her two‐dimensional model (Fraser 1996) which she later extended to take account of wider political forces (Fraser 2005). Fraser's three constituents of social justice—representation, recognition and redistribution ‐ occur across economic, cultural and political spheres (Fraser 2005; Hölscher 2014). One is not undermined by the other and they are often intertwined in everyday life (Fraser 1998). Ultimately social justice relies on mutual reinforcement of all dimensions and is about participatory parity: what does it take for all to participate on an equal footing in social life? In this article we explore the extent to which Brexit has impacted on women's recognition, redistribution and representation.

Representation refers to the political sphere, involving electoral representation as well as political agenda setting. What questions are asked and how boundaries of inclusion and exclusion are drawn are important considerations. The latter aspects encompass participatory parity within the nation state while also questioning the territorial state as the unit of justice (Gomes 2018). Historically this matter has resonance in the context of Northern Ireland and the involvement of women in different spheres of the peace process, not least through the Women's Coalition. Representation also relates to the question of who influences decision making processes. Additionally, the way in which issues within a society are framed are of utmost importance; mis‐framing representing a particular form of injustice (Fraser 2005). In thinking through processes of representation two key questions arise. Firstly, ‘do the boundaries of the political community wrongly exclude some who are actually entitled to representation?’ and secondly, ‘do the community's decision rules accord equal voice in public deliberations and fair representation in public decision‐making to all members?’ (after Taylor 1994).

For Fraser, redistribution is connected to socioeconomic justice. It does not occur where there is economic marginalisation, exploitation and deprivation as these injustices mean that people cannot participate equally with others, be that in the labour market or within education systems. For instance, an individual working in the low wage economy which demands long working hours, may have less time to participate in wider community and family activities when compared to white collar workers. Likewise, Fraser (2005) describes how institutionalized hierarchies of cultural value can deny others social standing and impede cultural recognition.

Redistribution is closely connected to recognition as one can lead to the other. Fraser's conceptualisation of recognition affords individuals the status needed to participate fully and equally in society. Misrecognition occurs through ‘institutionalized patterns of cultural value [that] constitute some actors as inferior, excluded, wholly other or simply invisible, hence as less than full partners in social interaction’ (Fraser 2001, 24). According to this definition, the Irish have historically been misrecognised by the English. As well as the Irish being perceived as having value through providing a steady labour supply to England to do the jobs that others reject (Hickman and Ryan 2020), they have also been othered due to different religious identities and allegiances. This has resulted in unequal treatment, the prevalence of Irish stereotypes within the workplace and other settings, and the inability to enjoy full and equal access to services for example housing (Ryan 2007; Hickman and Ryan 2020).

Misrecognition is connected to cultural aspects and relates to cultural domination, non‐recognition and disrespect.^3^ Taylor goes so far as to argue that it is a vital human need, claiming that when we are not recognised we can suffer ‘real damage, real distortion, if the people or society around them mirror back to them a confining or demeaning or contemptible image of themselves’ (1994, 25). Although misrecognisition has a very individualised impact, Fraser makes it clear that the issues underpinning social justice transcend individual failings, they are not about psychological shortcomings (Hölscher 2014). Instead, all elements are intertwined and are fundamental within a wider social justice framework (Fraser 2005). Thus, it is not about a question of more emphasis on one pillar over another such as redistribution at the expense of recognition, that is class politics over identity politics. Fraser asserts that the state has a clear role in upholding social justice, drawing attention to how the interests of individuals and communities are represented within the public sphere and how this necessarily involves the state (in different forms) and is distinct from economic and cultural aspects.

## 2 Materials and Methods

This research comprised qualitative social research, conducted between December 2022 and May 2023. The research was commissioned by The Equality Commission for Northern Ireland to explore the actual, potential and perceived impact of Brexit on women. Ethics approval was granted by Newcastle University. Along with a review of grey and academic literature, primary data collection was carried out. This comprised an expert seminar with representatives from women's organisations across Northern Ireland; six focus groups with targeted groups (representing women from minority ethnic communities, rural women, women living in border communities; women with disabilities; women from disadvantaged communities; women with caring responsibilities; women from different community backgrounds and LGBTQ women); and 11 interviews with key stakeholders. Our research design was thus guided by an intersectional understanding, recognising that women are far from a homogenous group (Crenshaw 1991). A visual artist was commissioned to produce visual minutes which were used to communicate our research to a wider audience. A small steering group with key representatives from the women's sector ensured connection to ongoing research and policy developments.

We accessed participants for the expert seminar through key contacts, while focus group participants were recruited through third sector organisations and researcher contacts. Finally, snowball sampling from the expert seminar was used to identify interviewees. The data generated was digitally recorded and transcribed with assistance from Otter.ai software. We then undertook preliminary coding to understand the actual, perceived and potential impact of Brexit on women in Northern Ireland. This was an iterative process, with the research team coding the expert seminar at the same time as convening interviews and focus groups. Thus, the emerging sub‐themes informed discussion points and interview questions. The research team discussed emerging findings during several review sessions before finalising the analysis. We proceed with an analysis of legal and policy frameworks for gender equality in Northern Ireland, before going on to consider the findings from our primary data collection.

### 2.1 Where Are the Women? Representation, Brexit Negotiations and Beyond

The UN Women, Peace and Security (WPS) agenda is highly relevant to Northern Ireland as a post‐conflict society, particularly given the destabilising impact of Brexit on the Belfast (Good Friday) Agreement (Pierson 2019). Its emphasis on the protection and participation of women in public life is crucial, as protection enables women to engage fully in the public sphere (Turner and Swaine 2023), including in dialogue, debate, and consultation on Brexit. Yet we found a tangible fear amongst women on speaking about Brexit either at home, but particularly in the public sphere:

It's not black and white. So, you know, just like, how can we create, and how can we ask politicians to create safe spaces, for those of us with, that have different concerns to put them on the table?

(Focus Group 6)

And I think, I don't want to be pessimistic, but like, like Northern Ireland isn't really the most peaceful place. So it can easily erupt and I think women will definitely suffer first, whether it's domestic violence during conflict [if it] becomes worse, whether it's paramilitary, sort of paramilitaries being empowered again, and being back to the streets, not that they ever disappeared.

(Focus Group 5)

In addition to the significant barriers that prevent women from fully participating in Brexit‐related dialogue, key actors failed to meaningfully consult with women. Women were not recognised as being full and legitimate partners in the Brexit negotiations with evidence of ‘policymakers at a local, UK Government and EU level wishing to consult with Northern Ireland stakeholders in relation to the Protocol are doing so on an ad hoc basis, with no readily transparent or structured process’ (Centre for Cross Border Studies (CCBS), n.d.). The ad hoc nature of this process included poor quality relations which are not respectful of busy diaries:

You get a request on a Monday saying can you come to a meeting at four o'clock on Wednesday, assuming we have nothing else to do, but then… sometimes it gets cancelled, again, with very short notice.

(Interview 6)

There was little to suggest that critical actors working for gender equality were effectively considered in the design of the negotiations and in areas of policy cooperation after the UK's withdrawal from the EU. This is evident in our broader analysis, where women's concerns about ‘everyday’ issues, such as access to healthcare, cross‐border travel, rising racism, violence against women, and legal protections, were largely ignored and not prioritised during the Brexit negotiations. As one interviewee explained:

…what we've basically been saying [is] ‘where's the woman's perspective in any of this?’ ‘How do you engage with women?’ And even beyond that ‘how do you engage with people on the ground?’ Because I think certainly …over the last year when the discussions of the Protocol were heaviest, there was that sense, certainly [among] women that things are happening *to* them, and not *with* them. And that was creating additional tension.’

(Interview 5)

Incorporating women's perspectives into negotiations through consultation is not a binary choice between engaging with women or addressing the concerns of unionists, nationalists, or other communities in Northern Ireland; rather, it is a fundamental component of fostering inclusive and equitable dialogue in a post‐conflict society. Failure to actively consult with women risks perpetuating a default framework in which different, often intersectional, identities and lived experiences are primarily understood through a narrow, male‐dominated perspective.

Where the women's sector was consulted, they represented a small proportion of those the UK and EU sought to engage with. There was some ad hoc engagement from the UK Government with the third and women's sectors, but not in a sustained manner and nor was it taken seriously. For example, an ESF (European Social Fund) user group that was in existence before Brexit, from 2019 became focussed on the replacement of EU funds. Convened by a third sector organisation, it also facilitated consultation with the UK government (post Brexit). Two quotes from two interviewees illustrate:

So [the Secretary of State] did come and engage with that group kind of, basically, it was an open invitation…they did do about three engagements with that group. It's probably the only significant kind of consultation they did with our sector via that group.

(Interview 3)

[policy makers are] just doing this tickbox consultation. But it's kind of worse than that, isn't it, that there isn't even any kind of resourcing for you to support members or even go to members and work out what Brexit means to them.

(Interview 6)

Another respondent talked about the importance of having social engagement in negotiations, which is found in other parts of Europe, arguing that if that had been part of the Brexit process things might have been very different:

… maybe would have been done better… it's no good just to say that, yeah, let's set up this group and this, blah, blah, blah, co‐design. It has to be…properly done, and particularly for women's sector organisations, and small organizations, there has to be resources, so that people can positively participate. And, you know, think about things such as childcare costs, travel costs, and all of those things that mean that women can genuinely participate and not just, you know, ‘these are the two women in Northern Ireland who, you know, represent all women's voices'…’

(Interview 7)

Mis‐framing occurs if some people are excluded from participation due to the way boundaries are drawn around an issue (Fraser 2005). The ‘gerrymandering’ of the political space by the territorial state, at the expense of those ‘poor and despised’ also prevents the oppressed from challenging their oppressors (Fraser 2010, 20). The issues to be discussed in the Brexit negotiations and subsequent EU‐UK relations demonstrate a clear case of mis‐framing with a near exclusive focus on trade and related issues. The ‘everyday’ issues concerning women, including healthcare, cross‐border travel, rising racism, violence against women, and legal protections were sidelined. Ongoing and evolving relations between the EU and the UK are discussed at the UK level through different bodies including the EU‐UK Civil Society Forum and the Domestic Advisory group. To become involved with these groups, prospective participants must submit an expression of interest:

…expressions of interest were invited. And then, you know, appointments were made to the bodies in both cases.

(Interview 3)

The agenda for the Civil Society Forum meeting in November 2023 included four main items which were focussed on trade in goods; regulatory cooperation; energy and climate change; and trade in services. Meanwhile during Brexit negotiations, despite the prominence of equality issues within the GFA, consideration of the gendered impact of Brexit was absent from negotiations on both the UK and EU sides (Guerrina et al. 2020). Just as gender issues were sidelined, trade issues were front and centre of negotiations (Wright et al. 2024a)—the political space was determined by territorial states—the EU and the UK government. Both the UK and EU prioritised meetings with corporate lobbyists, at the expense of the third sector (ibid).

Meanwhile a significant lack of transparency around negotiations made it difficult to know who attended and what was discussed, with both UK and EU decision makers refusing to release participant lists, agendas and minutes (Corporate Europe Observatory and Global Justice Now 2017). These dynamics contributed to the creation of an information vacuum which in turn created space for misinformation and uncertainty:

…as a service provider that works with the grassroots, with community groups, the extent to which we have to counter misinformation and misconceptions around what people's rights are is really quite troubling… In tight knit migrant communities this becomes increasingly the case, the more kind of disconnected from the system [they are], a community has lower levels of English language and lower levels of knowledge of how to navigate the system, you would have instances of gatekeepers, basically, using information in ways that leverages their own power within the communities.

(Expert Seminar)

…any vacuum here leaves the space for kind of some kind of negative outcome. I mean, what we're seeing at the minute in the context of the Windsor framework is that the vacuum around decision making, so the framework was published, you know, weeks ago, now…then there's a vacuum and what happens in the vacuum is that media amplify division.

(Interview 2)

The protracted Brexit negotiations, including the way in which Brexit was framed, designed and defined, was such that women's voices were marginalised. The tools and techniques to achieve this were wide ranging, including the lack of transparency; the creation of asymmetric power relations; and the privileging of particular groups and voices over others. Fundamentally parity of participation was absent, this being a core component of social justice.

### 2.2 Women's Voices Within ‘Democratic Iterations’

The European Union (EU) was considered by those advocating for Brexit as a revolt against the so‐called elites (Calhoun 2016) reflecting the belief that transnational rights and human rights are in contradiction with one another and a lack of understanding of what sovereignty means in the 21st century (Benhabib 2016). As Benhabib argues ‘[s]overeignty can no longer be understood as the supreme power of a single political authority over all that is living and dead on its territory’ (2016, 113), not least due to the range of international agreements such as the 1948 Universal Declaration of Human Rights. The protracted Brexit process involved the entanglement of politics and law whereby there was a failure of politics to produce the shared norms that Post (2010) argues are then enforced by the law. Consequently, politics and the law around Brexit are complex and increasingly challenged because Brexit created contestations about the limits of both law and politics since then we have seen how, in the words of Post, ‘judicial judgements engage but do not pre‐empt politics’ (2010: 1347).

The EU as a guarantor of the GFA placed it front and centre of negotiations concerning the UK's exit from the EU (Brexit), because the agreement was premised on both the UK and Ireland being members of the EU (Doyle and Connolly 2019). Thus, within the EU‐UK Withdrawal Agreement, Protocol Article 2 sets out the UK government's commitments on equality and human rights post Brexit. Protocol Article 2 relates to human rights and equality protection within the GFA and is underpinned by EU law that exists on or before 31 December 2020 (ECNI and NI Human Rights Commission 2022). As already mentioned, Protocol Article 2 commits the UK government to upholding the rights, safeguards and equality of opportunity as set out in Chapter Six of the Belfast (Good Friday) Agreement. The UK Government has also committed to ensuring that some of Northern Ireland's equality laws will keep pace with any future changes to certain EU equality laws (ECNI and NI Human Rights Commission 2022).^4^ The Protocol further requires that the UK government facilitates the work of relevant bodies, for example Northern Ireland Human Rights Commission (NIHRC), Equality Commission for NI (ECNI), Joint Committee of representatives of the Human Rights Commissions of Northern Ireland and Ireland and the Irish Human Rights and Equality Commission (IHREC) to uphold human rights and equality standards. This means that some new and emerging EU legislation, such as those on carers' leave, flexible working or stronger protections for new mothers, lie outside this duty and there is a danger that human rights will be sidelined (ECNI 2022). The implications of dynamic alignment, keeping pace and equivalence are not fully understood but have generated high levels of uncertainty and indeed fear about what the future will hold (Wright et al. 2024b). One interviewee elaborates:

… will we be able to positively influence Employment Bills, so that, for example, it contains some of the measures, which are now going to have to be transposed under the adequate minimum wages directive in the Republic, which also includes minimum standards around collective bargaining… I do genuinely feel for the civil servants who are having to work not only in this vacuum, but also, you know, grappling with this whole retained EU law stuff

(Interview 7)

Legal scholars argue that certainty in the law is likely to emerge only through interpretation of the law, which means courts will be drawn in to provide clarity (Craig et al. 2022). This is a good example of what Benhabib labels ‘democratic iterations’ in referring to ‘complex processes of public argument, deliberation and exchange’ whereby rights are debated and contested and within legal and political institutions and via civil society (2016, 122). Such democratic iterations are necessary to contribute to processes of interpreting rights claims (Benhabib 2016). This process is highly pertinent for Northern Ireland given the complexity of the process as outlined earlier in this section and as these interviewees explain:

… people have heard so many different versions of what Brexit means for Northern Ireland, that on a day‐to‐day basis [it is] just jumbled up in people's heads. But more than that, I think it's even jumbled up in professional legal circles’ heads…until there are some cases that come through, some people that are willing to experiment with this and try to make it work, there's not going to be a widespread understanding of how Article 2 works, even within legal circles in NI…

(Interview 9)

… it's so complicated. You know, getting to grips with what no diminution means getting to grips with what keeping pace means

(Interview 7)

It is worth highlighting how when we approached one grassroots women's group to do a focus group, they initially responded by indicating that they had little to no knowledge of Brexit. Others talked about how men had told them that Brexit was too complex for them to understand. Brexit negotiations certainly created a major distraction from everyday issues as another woman explains:

…those resources are getting used up talking about Brexit means that they're not talking about things like gender equality, or, you know, all these other issues that we need to see progress on, like healthcare.

(Focus group 6)

Many social inclusion strategies were stalled due to the political impasse arising over Windsor Framework negotiations and include public consultation on the following strategies: Gender Equality, Disability, Sexual Orientation and Anti‐Poverty (Wright et al. 2024b). These are critical for addressing key inequalities facing women across childcare; employment; participation in political and public life; health and social care; and education.

Democratic iterations do not occur solely in courts and legislatures, increasingly they involve the participation of NGOs, producing expert reports and mobilising public opinion (Benhabib 2016, 122). Thus, the question of the recognition and capacity of advocacy groups, including those advocating for migrants, women and the LGBTQI + community, is highly relevant if we are to understand the process for contributing to deliberations around rights and legal obligations.

### 2.3 Redistribution of Resources: Funding for Section 75 Groups

The way in which Brexit impacted on individual women was notable, particularly for those on low incomes and those with migrant status. One interviewee described how her organisation had lobbied on behalf of different individuals to have their welfare payments backdated, noting the knock‐on implications for their health and wellbeing. She went on to explain:

…even for people who are exempt under any interpretation of the legislation, they are still being charged because the actions of the Health Trust seems in many cases to charge by default, like almost like an assumption that because this person has an immigration status that isn't ‘citizen’, they are liable to be charged…I should mention that, like most of the time when we're seeing this, it's related to maternity care or women's healthcare.

(Interview 8)

Prior to Brexit, funding from the European Union supported numerous third sector organisations in Northern Ireland, with many beneficiaries being Section 75 groups (as identified in the Northern Ireland Act 1998), including women, people with disabilities, minority ethnic individuals and young people (ECNI 2022). For example, the Women and Peacebuilding: Sharing the Learning Project was funded through PEACE III and its aim was to give a voice to women living in communities recovering from the worst impact of the conflict (McWilliams and Kilmurray 2015). That project raised issues of misrepresentation as it showed how peace was negotiated behind closed doors. The Northern Ireland Executive has a long history of administering EU funds in a targeted way that largely benefits Section 75 groups—although it is also noted that some of those funding streams have consistently overlooked Section 75 groups, especially women (McAreavey 2022). As noted in one of our interviews:

But the reality is that there hasn't been any real EU funding for gender equality for a long time… [but] there's obviously lots of other things around capacity building and maintaining the community and voluntary sector that indirectly provides mechanisms for women to get involved

(Interview 5)

However, other women talked about how as immigrants they personally benefited from the social support via projects that were funded by the EU which helped her with childcare. One woman explains how having access to childcare allowed her:

…go to Tesco or go shopping quickly and come back. That's gone. And if you are caring for a disabled child, every little helps… you could get that support, you could relax, and somebody can look after him because I don't have a mother….I don't have any brothers or sisters. So that help was very important. But now that funding is gone. Another issue I have seen within our communities that like, I'm actually a Ghanaian

(Focus Group 2)

Some EU funds were superseded by the UK's Shared Prosperity Fund, Community Renewal Fund and the Levelling Up Fund. Concerns were raised about the centralised way in which they were to be administered in Northern Ireland, that is directly by the then government department, the Department for Levelling Up, Housing and Communities (DLUHC) (since July 2024 renamed by the Labour government to the Ministry of Housing, Communities and Local Government (MHCLG). It was argued that this would fail to tap into local knowledge and expertise and would not secure vital cross‐community support in Northern Ireland (Nice et al. 2021), raising further issues in terms of support for Section 75 groups, as one of our interviewees describes:

And I think the big issue is that there's going to be a lot more competition for much more limited funds. And that's going to have an impact on the organisations because of finance … as long as I've been in the sector … just when you've started doing something, the funding runs out… there's a risk there’s going to be a lot more competition. When in actual fact, we need to work more collaboratively than ever…

(Interview 5)

In contrast to the seven‐year cycle for EU funds, the Shared Prosperity Fund (SPF) follows a three‐year cycle. This is problematic as it does not take account of the time taken to develop meaningful relationships, especially when working in communities where there are complex issues to be addressed and where trust can be in deficit (Byrne et al. 2009; Campbell et al. 2010). The lack of appreciation of complexity is reflected in original SPF documentation, where generic comments are made about targeting marginalised groups (O'Connell and Cunningham 2022). Another problem relates to the alleged lack of transparency in the way in which the funds were allocated in Northern Ireland and lack of information about equality screening and specifically how Section 75 legislation would be addressed (Hansard 2023). Concerns about the operations of the funds were expressed by a Northern Ireland government minister (Brien 2022), reflecting a demonstrable clash whereby territorial state action manipulates, or to rephrase Fraser (2010), gerrymanders, political space at the expense of marginalised social groups.

Fraser (1997) argues that political‐economic restructuring can resolve economic injustices. O'Connell and Cunningham (2022) recommend that, given the experiences of existing government departments in Northern Ireland in dealing with the local context including Section 75 requirements, the SPF ought to be delivered through existing structures in Northern Ireland for adequate monitoring and accountability. They argue that ‘it is difficult to establish the impact of the funding decisions on equality of opportunity and good relations in Northern Ireland’ (O'Connell and Cunningham 2022, 10). Although the full implications of a new funding regime for women in Northern Ireland are not fully understood (McAreavey 2022), more immediate economic injustices relate to the women's sector in Northern Ireland. Local media reported how a Women's Centre in Ards and north Down, supporting women through childcare; health and wellbeing services would not have its £900,000 EU funding replaced (Haslam 2023). The gap between EU and the replacement funds resulted in profound uncertainty for women's groups and the loss of expert staff and knowledge (see also Hansard 2023). This has serious implications for the delivery of services for women but also for the capacity of the women's sector to contribute to legal processes to help ‘to judge the legitimacy of a range of variation in the interpretation of a rights claim’ (Benhabib 2016, 123).

Claire Hanna MP, asked questions in Westminster about the problems arising from the central allocation of Shared Prosperity Funds within Northern Ireland. She also raised concerns about projects supporting women, young people and minority ethnic communities, with reports later made at an all‐Island Women's Forum of the closure of women's centres.^5^ Women's spaces are important: women's centres provide essential safe spaces for conversation, and this is especially pertinent for those who have few opportunities to exchange views and engage in discussion on critical issues.

### 2.4 Recognising and Upholding Rights in a Post‐Conflict Society

Different layers of governance impact on gender equality in Northern Ireland. In this section we review how different bodies, including the EU and the UK, recognise the rights of women, and their intersecting identities in Northern Ireland. There was a clear sense of a loss of rights for women in their everyday lives, including the above cited access to healthcare, other women feared for the rollback of rights in the workplace. We found evidence of misrecognition of women based on their sexual identity:

…as a queer woman whose not from [Northern Ireland] even though I'm not an expert in the politics here…[Brexit is] definitely not in my favour in any way…I think Northern Ireland has always been at the bottom of the list when it comes to [Westminster Ministers]

(Focus group 5)

She discussed how the rhetoric of Unionist political parties in Northern Ireland ‘is against my existence’. This raises the question of how the rights of LGBTQI + groups would be upheld. One woman explained how healthcare was impacting on women:

I work in perinatal mental health. So you know, there's no mental health strategy, there's funding for this year… we're campaigning for a mother and baby unit, but at the end of the day, we know that the health service has been under such pressure. So that has consequences for women …and that's all as a consequence of the assembly collapsing and the protocol…this has become the most recent divide to divide our communities here.

(Focus Group 6)

We found that certain groups of women experienced greater impacts than others, struggling to retain autonomy over their everyday lives. For instance, during one of our focus groups this woman explained:

I’m not from here, I’m from Syria…I think women will definitely suffer first, whether it's domestic violence…whether it's paramilitaries being empowered again, and being back to the streets, not that they ever disappeared. I think that Brexit will see women suffering first, and you see it as well, again, when it comes to the hospital, whether it's abortion, whether it's IVF, it's like everything is behind here to begin with.

(Focus Group 5)

Unlike the rest of the UK where the Equality Act prevails, there is no consolidated, harmonised, single equality legislation. Gaps include no provision for reporting on the gender pay gap; no provision for intersectional discrimination; and no protection against discrimination by private clubs (Black and Doherty 2024). In other words, women in Northern Ireland are not recognised as being equal to women elsewhere in the UK and thus some women are not given the same protection as in the rest of the UK. For instance, the historical absence of a Violence Against Women Strategy in Northern Ireland resulted in the region not receiving comparable levels of funding to address this issue, unlike other UK nations. This gap was finally addressed in 2024 with the adoption of such a strategy. The delay was particularly concerning given that Northern Ireland has the highest levels of gender‐based violence compared to the other UK nations (Wright et al. 2024a). The lack of funding has an impact on the UK's ability to uphold its obligations in Northern Ireland to legal frameworks that were created to protect women against domestic abuse, including the European Protection Order and the European Victims' Rights Directives. Given that incidences of domestic abuse rose dramatically during the pandemic (Women’s Aid Federation for Northern Ireland 2020) and the legacies of gender‐based violence arising from the conflict in Northern Ireland (Swaine 2022), the matter is highly relevant for women's everyday lives.

## 3 Conclusions

We argue in this article that Brexit does little to contribute to social justice for citizens in Northern Ireland and different tactics have resulted in the exclusion or marginalisation of women from meaningful consultation. Brexit has redistributed resources, effectively sidelining the interests of Section 75 groups. This is exclusionary per se and it is a blatant bypassing of international legislation enshrined in the Belfast/Good Friday Agreement and according to stated commitments to implement the UN Women, Peace and Security agenda.

The fundamental misunderstanding by Westminster politicians of the international border between Ireland and Northern Ireland that Hickman and Ryan write about, has been ably demonstrated by the prolonged Brexit negotiations, particularly around the Northern Ireland Protocol and the subsequent Windsor Framework. Pro‐Brexit politicians paradoxically chose to overlook that uniqueness; in so doing they disregarded the intricacies of the Good Friday Agreement and the underpinning assumption that the UK would remain part of the European Union (EU). Such brutal treatment of a peace agreement is a flagrant disregard for the residents of Northern Ireland, reflecting how the Irish Question remains an ‘irritant’ for the UK government (Gilligan 2017, 5) and demonstrating a profound lack of understanding of critical relations between Northern Ireland and surrounding jurisdictions. It also demonstrates tension between international obligations and national sovereignty, suggesting that the UK government has reverted to a historic or Westphalian understanding of territorial sovereignty and, with that, sovereign immunity (after Benhabib 2016). The degree to which this will prevail in the future warrants further research.

There remain very many challenges in relation to the socio‐economic impact of the UK's withdrawal from the EU on women and girls. These include, but are not limited to, discrimination on the basis of gender, race, disability and ethnicity; access to health services; child sexual exploitation; increased levels of stress among farm families; employment rights; and domestic abuse.^6^ We have shown how these challenges impact on different groups in different ways through an intersectional lens. Immigrant women have experienced particular misrecognition and the redistribution of resources is likely to impact greatly on women from lower income families as well as immigrant women who have fewer social networks. The magnitude of these impacts are not yet fully understood.

Our research draws attention to the misrecognition of women as equal participants in the political process that was Brexit. At first glance, this may seem surprising, considering the active involvement of women in the peace process during the late 1990s. However, this aligns with the understanding that simply ‘adding women and stirring’ into peace processes is insufficient. The deeply entrenched masculinised norms that shape these processes, including in Northern Ireland, often limit their transformative potential, leaving existing gender hierarchies intact (McAuliff 2022). Unsurprisingly then, the same patterns were evident during consultations on the Brexit negotiations, where gender equality actors who were invited to participate were included as part of a broader, largely superficial attempt to engage with civil society organisations. More broadly, women in our study expressed concern with everyday issues, including health, housing and employment. It is notable that these matters resonate with the issues that women successfully negotiated for in the GFA and that ultimately made it more relevant to people's lives.

The manipulation of political and territorial boundaries during the Brexit process has resulted in the exclusion of certain voices, such as Section 75 groups due to the way in which replacement funds are to be distributed directly by the UK government. This complex interconnection between distribution, recognition and representation shows how the achievement of social justice requires action and cooperation from different interests including political leaders, policymakers, and third sector organisations.

Gomes's (2018) reminder of Benhabib's (2016) work is instructive. At many times in Brexit negotiations relations between the UK and the EU as negotiating partner were fragmented. Our research has demonstrated little evidence of critical dialogue between the national and international sphere, due for instance to the focus on trading relations. This was evident in the failure of the UK and EU to uphold their commitments to implementing the UN Women, Peace, and Security agenda, which would have prioritised meaningful consultations with women. Critical national and international engagement is something that Benhabib (2016) strongly advocates if we are to create space for voice and representation of those on the margins. Promoting the human rights of all citizens in Ireland requires positive engagement of and interaction between a range of national and international bodies. Only through commitment to this endeavour will social justice be achievable, at which point all citizens will enjoy access to fair resources as well as being fully recognised and represented within society.

## Ethics Statement

Ethics approval was granted by Newcastle University (25314​​/2021). The material is the authors' own original work and has not been published elsewhere, nor is it currently being considered for publication elsewhere. Informed consent was secured from all participants. The research was governed by the Research Ethics policy of Newcastle University.

## Conflicts of Interest

The authors declare no conflicts of interest.

## Data Availability

Research data are not shared.

## Endnotes

^1^
Sovereigntism is understood to be part of a ‘growing resistance to the force of transnational law in the contemporary world’ (Benhabib 2016, 111).

^2^
We recognise race as a socially constructed category that can lead to racist behaviour towards a person or group of people who belong to the same group. As Ryan and Hickman and others argue, the racialization of the Irish in England is so ingrained in everyday culture as to render it invisible (see also Walter 2017). Thus the Irish have long been the butt of jokes based on their accent and supposed stupidity.

^3^
This differs from how other social theorists conceive of recognition, notably Honneth (2003),  (2005) and Taylor (1994) who view it as being inherent to the self whereby the lack of recognition is damaging the individual.

^4^
Council Directive 2004/113/EC of 13 December 2004 implementing the principle of equal treatment between men and women in the access to and supply of goods and services; Directive 2006/54/EC of the European Parliament and of the Council of 5 July 2006 on the implementation of the principle of equal opportunities and equal treatment of men and women in matters of employment and occupation; Council Directive 2000/43/EC of 29 June 2000 implementing the principle of equal treatment between persons irrespective of racial or ethnic origin; Council Directive 2000/78/EC of 27 November 2000 establishing a general framework for equal treatment in employment and occupation; Directive 2010/41/EU of the European Parliament and of the Council of 7 July 2010 on the application of the principle of equal treatment between men and women engaged in an activity in a self‐employed capacity and repealing Council Directive 86/613/EEC; Council Directive 79/7/EEC of 19 December 1978 on the progressive implementation of the principle of equal treatment for men and women in matters of social security.

^5^

https://www.nwci.ie/learn/article/loss_of_eu_funding_has_devastating_consequences_for_womens_sector_in_ni.

^6^
NI Human Rights Commission, Annual Report of the NIHRC and the ECNI on the implementation of Protocol Article 2 2021–2022.

## References

[bjos13207-bib-0001] Achilleos‐Sarll, C. , and B. Martill . 2019. “Toxic Masculinity: Militarism, Deal‐Making and the Performance of Brexit.” In Gender and Queer Perspectives on Brexit, edited by M. Dustin , N. Ferreira , and S. Millns , 15–44. Palgrave‐Macmillan.

[bjos13207-bib-0002] Aggestam, K. , and A. Towns . 2018. “The Gender Turn in Diplomacy: A New Research Agenda.” International Feminist Journal of Politics 21, no. 1: 9–28. 10.1080/14616742.2018.1483206.

[bjos13207-bib-0003] Bachelet, M. 2015. “Women as Agents of Peace and Stability: Measuring the Results in Clinton, HR, Rodham.” In Women on the Frontlines of Peace and Security, edited by L. Panetta , V. Amos , M. Bachelet , et al, 95–112. National Defence University Press. https://digitalcommons.ndu.edu/cgi/viewcontent.cgi?article=1009&context=books‐and‐book‐chapters.

[bjos13207-bib-0004] Bagehot . 2018. “The Elite That Failed.” Economist. https://www.economist.com/britain/2018/12/22/the‐elite‐that‐failed.

[bjos13207-bib-0005] Benhabib, S. 2016. “The New Sovereigntism and Transnational Law: Legal Utopianism.” Democratic Scepticism and Statist Realism 5, no. 1: 109–144: Global Constitutionalism. 10.1017/s2045381716000010.

[bjos13207-bib-0006] Black, N. , and G. Doherty . 2024. Comparative Study of Equality Legislation in the United Kingdom and Ireland, 125–2024. Northern Ireland Assembly: Research and Information Service Briefing Note Paper 29/24, NIAR.

[bjos13207-bib-0007] Brien, P. 2022. The UK Shared Prosperity Fund. Research Briefing, UK Parliament House of Commons Library. https://commonslibrary.parliament.uk/research‐briefings/cbp‐8527/.

[bjos13207-bib-0008] Byrne, S. , O. Skarlato , E. Fissuh , and C. Irvin . 2009. “Building Trust and Goodwill in Northern Ireland and the Border Counties: The Impact of Economic Aid on the Peace Process.” Irish Political Studies 24, no. 3: 337–363. 10.1080/07907180903075801.

[bjos13207-bib-0009] Calhoun, C. 2016. “Brexit is Mutiny Against the Cosmopolitan Elite.” New Perspectives Quarterly 33, no. 3: 50–58. 10.1111/npqu.12048.

[bjos13207-bib-0010] Campbell, A. , J. Hughes , M. Hewstone , and E. Cairns . 2010. “Social Capital as a Mechanism for Building a Sustainable Society in Northern Ireland.” Community Development Journal 45, no. 1: 22–38. 10.1093/cdj/bsn033.

[bjos13207-bib-0011] Centre for Cross Border Studies (CCBS) (n.d.) Briefing to Committee for Executive Office. Armagh, CCBS. Accessed July 8, 2024. https://crossborder.ie/newsite/wp‐content/uploads/2021/12/CCBS‐Briefing‐to‐the‐Committee‐for‐the‐Executive‐Office‐291121.pdf.

[bjos13207-bib-0012] Corporate Europe Observatory and Global Justice Now . 2017. “Big Business Britain Part Two: How Corporate Lobbyists Dominate Secret Meetings With Brexit Negotiators in London and Brussels Corporate Europe Observatory.” Briefing Paper, July. https://corporateeurope.org/sites/default/files/big_business_britain_2_0.pdf.

[bjos13207-bib-0013] Craig, S. , A. Deb , E. Frantziou , et al. 2022. European Union Developments in Equality and Human Rights: The Impact of Brexit on the Divergence of Rights and Best Practice on the Island of Ireland. NI Human Rights Commission and Equality Commission.

[bjos13207-bib-0014] Crenshaw, K. 1991. “Mapping the Margins: Intersectionality, Identity Politics, and Violence Against Women of Color.” Stanford Law Review 43, no. 6: 1241–1299. 10.2307/1229039.

[bjos13207-bib-0015] Crenshaw, K. 2017. “Kimberlé Crenshaw on Intersectionality, More Than Two Decades Later.” Colombia Law School: 8 June 2017. https://www.law.columbia.edu/pt‐br/news/2017/06/kimberle‐crenshaw‐intersectionality.

[bjos13207-bib-0016] Doyle, J. , and E. Connolly . 2019. “The Effects of Brexit on the Good Friday Agreement and the Northern Ireland Peace Process.” In Peace, Security and Defence Cooperation in Post‐Brexit Europe, edited by C. A. Baciu and J. Doyle , 79–95. Springer.

[bjos13207-bib-0017] ECNI 2022. Impact of Brexit on Section 75 Equality Groups in Northern Ireland: EU Funding. Policy Recommendations.

[bjos13207-bib-0018] ECNI and NI Human Rights Commission 2022. Annual Report of the NIHRC and the ECNI on the Implementation on of Protocol Article 2. ECNI and Human Rights Commission.

[bjos13207-bib-0019] Fagan, C. , and J. Rubery . 2018. “Advancing Gender Equality Through European Employment Policy: The Impact of the UK’s EU Membership and the Risks of Brexit.” Social Policy and Society 17, no. 2: 297–317. 10.1017/s1474746417000458.

[bjos13207-bib-0020] Fraser, N. 1996. Social Justice in the Age of Identity Politics: Redistribution, Recognition and Participation, *the Tanner Lectures on Human Values* . Stanford University. https://tannerlectures.utah.edu/_resources/documents/a‐to‐z/f/Fraser98.pdf.

[bjos13207-bib-0021] Fraser, N. 1997. Justice Interruptus: Critical Reflections on the ‘Postsocialist’ Condition. Routledge.

[bjos13207-bib-0022] Fraser, N. 1998. Social Justice in the Age of Identity Politics: Redistribution, Recognition, Participation, *WZB Discussion Paper*, No. FS I 98‐108. Wissenschaftszentrum Berlin für Sozialforschung (WZB).

[bjos13207-bib-0023] Fraser, N. 2001. “Recognition Without Ethics?” Theory, Culture & Society 18, no. 2–3: 21–42. 10.4135/9781446216897.n2.

[bjos13207-bib-0024] Fraser, N. 2005. “Reframing Justice in a Globalizing World.” New Left Review 36: 31.

[bjos13207-bib-0025] Fraser, N. 2010. Scales of Justice—Reimagining Political Space in a Globalizing World. Columbia University Press.

[bjos13207-bib-0026] Galpin, C. , and P. Vernon . 2023. “Post‐Truth Politics as Discursive Violence: Online Abuse, the Public Sphere and the Figure of ‘The Expert’.” British Journal of Politics & International Relations 26, no. 2: 423–443. 10.1177/13691481231202641.

[bjos13207-bib-0027] Gilligan, C. 2017. Northern Ireland and the Crisis of Anti‐Racism. Rethinking Racism and Sectarianism. Manchester University Press.

[bjos13207-bib-0028] Gilligan, C. 2021. “Methodological Nationalism and the Northern Ireland Blind‐Spot in Ethnic and Racial Studies.” Ethnic and Racial Studies 45, no. 3: 431–451. 10.1080/01419870.2021.1950793.

[bjos13207-bib-0029] Golec De Zavala, A. , R. Guerra , and C. Simão . 2017. “The Relationship Between the Brexit Vote and Individual Predictors of Prejudice: Collective Narcissism, Right Wing Authoritarianism, Social Dominance Orientation.” Frontiers in Psychology 8: 1–14. 10.3389/fpsyg.2017.02023. 29230185 PMC5712068

[bjos13207-bib-0030] Gomes, J. C. A. 2018. “Nancy Fraser’s Tridimensional Approach to Justice: Contributions and Provocations to the Practice of Domestic Litigators.” International Journal of Constitutional Law 16, no. 3: 1021–1024. 10.1093/icon/moy075.

[bjos13207-bib-0031] Guerrina, R. , T. Exadaktylos , and S. Guerra . 2018. “Gender, Ownership and Engagement During the European Union Referendum: Gendered Frames and the Reproduction of Binaries.” European Journal of Politics and Gender 1, no. 3: 387–404. 10.1332/251510818x15395099987095.

[bjos13207-bib-0032] Guerrina, R. , and A. Masselot . 2018. “Walking Into the Footprint of EU Law: Unpacking the Gendered Consequences of Brexit.” Social Policy and Society 17, no. 2: 319–330. 10.1017/s1474746417000501.

[bjos13207-bib-0033] Guerrina, R. , and H. Murphy . 2016. “Strategic Silences in the Brexit Debate: Gender, Marginality and Governance.” Journal of Contemporary European Research 12, no. 4: 872–880. 10.30950/jcer.v12i4.814.

[bjos13207-bib-0034] Guerrina, R. , K. A. M. Wright , and T. Haastrup . 2020. “Living up to the Women, Peace and Security Agenda? Gender Must Be a Core Element of Brexit Negotiations.” LSE Blog. https://blogs.lse.ac.uk/brexit/2020/02/19/living‐up‐to‐the‐women‐peace‐and‐security‐agenda‐gender‐must‐be‐a‐core‐element‐of‐brexit‐negotiations/.

[bjos13207-bib-0035] Hansard 2023. EU Funding: Northern Ireland. UK Parliament. https://hansard.parliament.uk/commons/2023‐02‐01/debates/BE836E3B‐9AA4‐4441‐960E‐0B4BDD4351D3/EUFundingNorthernIreland.

[bjos13207-bib-0036] Haslam, A. 2023. “European Social Fund: Groups Face Cuts to Services Despite £57m UK Cash.” BBC News. https://www.bbc.co.uk/news/uk‐northern‐ireland‐65123873.

[bjos13207-bib-0037] Hickman, M. J. , and L. Ryan . 2020. “The ‘Irish Question’: Marginalizations at the Nexus of Sociology of Migration and Ethnic and Racial Studies in Britain.” Ethnic and Racial Studies 43, no. 16: 96–114. 10.1080/01419870.2020.1722194.

[bjos13207-bib-0038] Hölscher, D. 2014. “Considering Nancy Fraser's Notion of Social Justice for Social Work: Reflections on Misframing and the Lives of Refugees in South Africa.” Ethics and Social Welfare 8, no. 1: 20–38. 10.1080/17496535.2012.744845.

[bjos13207-bib-0039] Honneth, A. 2003. “On the Destructive Power of the Third: Gadamer and Heidegger’s Doctrine of Intersubjectivity.” Philosophy & Social Criticism 29, no. 1: 5–21. 10.1177/0191453703029001830.

[bjos13207-bib-0040] Honneth, A. 2005. The Struggle for Recognition: The Moral Grammar of Social Conflicts. MIT Press.

[bjos13207-bib-0041] Hozić, A. A. , and J. True . 2017. “Brexit as a Scandal: Gender and Global Trumpism.” Review of International Political Economy 24, no. 2: 270–287. 10.1080/09692290.2017.1302491.

[bjos13207-bib-0042] Kilkey, M. , A. Piekut , and L. Ryan . 2020. “Brexit and Beyond: Transforming Mobility and Immobility.” Central and Eastern European Migration Review 9: 5–12.

[bjos13207-bib-0043] MacLeavy, J. 2018. “Women, Equality and the UK’s EU Referendum: Locating the Gender Politics of Brexit in Relation to the Neoliberalising State.” Space and Polity 22, no. 2: 205–223. 10.1080/13562576.2018.1502610.

[bjos13207-bib-0044] McAreavey, R. 2019. “Seasonal Workers Schemes: Can They Achieve Social Justice?” Europa XXI 37: 37–52: Polish Academy of Sciences. 10.7163/eu21.2019.37.3.

[bjos13207-bib-0045] McAreavey, R. 2022. Looking Back to Go Forward. A Review of Rural Development Funding in Northern Ireland. Rural Community Network and Rural Women’s Network.

[bjos13207-bib-0046] McAuliff, A. K. 2022. “Peace Negotiations as Sites of Gendered Power Hierarchies.” International Negotiation 28, no. 2: 176–200. 10.1163/15718069-bja10072.

[bjos13207-bib-0047] McWilliams, M. , and A. Kilmurray . 2015. “From the Global to the Local: Grounding UNSCR 1325 on Women, Peace and Security in Post Conflict Policy Making.” Women's Studies International Forum 51: 128–135. 10.1016/j.wsif.2014.11.006.

[bjos13207-bib-0048] Minto, R. , and A. Parken . 2021. “The European Union and Regional Gender Equality Agendas: Wales in the Shadow of Brexit.” Regional Studies 55, no. 9: 1550–1560. 10.1080/00343404.2020.1826422.

[bjos13207-bib-0049] Nice, A. , A. Paun , and D. Hall . 2021. The UK Shared Prosperity Fund. Strengthening the Union or Undermining Devolution. Institute for Government.

[bjos13207-bib-0068] Northern Ireland Act . 1998. UK Public General Acts, c. 47. https://www.legislation.gov.uk/ukpga/1998/47/contents.

[bjos13207-bib-0050] O’Connell, R. , and T. Cunningham . 2022. Impact of Brexit on Section 75 Equality Groups in Northern Ireland: EU Funding. ECNI.

[bjos13207-bib-0051] O’Connell, R. , F. Ní Aoláin , and L. Malagón . 2024. “The Belfast/Good Friday Agreement and Transformative Change: Promise, Power and Solidarity.” Israel Law Review 57, no. 1: 4–36. 10.1017/S0021223723000031.

[bjos13207-bib-0052] Pierson, C. 2019. “Gendering Peace in Northern Ireland: The Role of United Nations Security Council Resolution 1325 on Women, Peace and Security.” Capital & Class 43, no. 1: 57–71. 10.1177/0309816818818087.

[bjos13207-bib-0053] Post, R. 2010. “Theorizing Disagreement: Re‐Conceiving the Relationship Between Law and Politics.” California Law Review 98, no. 4: 1319–1350.

[bjos13207-bib-0054] Ryan, L. 2007. “Who Do You Think You Are? Irish Nurses Encountering Ethnicity and Constructing Identity in Britain.” Ethnic and Racial Studies 30, no. 3: 416–438. 10.1080/01419870701217498.

[bjos13207-bib-0055] Sanders, A. , and J. Flavell . 2023. “The Direction of Gender Equality Policy in Britain Post‐Brexit: Towards a Masculinised Westminster Model.” Journal of European Public Policy 30, no. 11: 2303–2325. 10.1080/13501763.2023.2200820.

[bjos13207-bib-0056] Swaine, A. 2022. “Resurfacing Gender: A Typology of Conflict‐Related Violence Against Women for the Northern Ireland Troubles.” Violence Against Women 29, no. 6‐7: 1391–1418. 10.1177/10778012221114923. 36006922 PMC10090524

[bjos13207-bib-0057] Taylor, C. 1994. “The Politics of Recognition.” In Multiculturalism: Examining the Politics of Recognition, edited by A. Gutmann , 25–73. Princeton University Press.

[bjos13207-bib-0058] The Guardian . 2024. “The Guardian View on the General Election in Northern Ireland: Time for London to Re‐Engage: Editorial.” Guardian. https://www.theguardian.com/commentisfree/article/2024/jun/30/the‐guardian‐view‐on‐the‐general‐election‐in‐northern‐ireland‐time‐for‐london‐to‐re‐engage.

[bjos13207-bib-0059] Turner, C. , and A. Swaine . 2023. “Law, Language and the Power of ‘Invisible Threats’ of Violence Against Women.” Journal of Law and Society 50, no. 3: 392–413. 10.1111/jols.12442.

[bjos13207-bib-0060] UK Government 2018. Taking Back Control of Our Borders, Money and Laws while Protecting Our Economy, Security and Union. EU Exit. Controller of Her Majesty’s Stationery Office: HM Government: CM9741.

[bjos13207-bib-0061] UK Government 2020. Protocol on Ireland/Northern Ireland (‘Protocol Article 2’) to the EU Withdrawal Agreement. UK Government. https://assets.publishing.service.gov.uk/media/5f2d14f7d3bf7f1b10d58f8c/Explainer__UK_Government_commitment_to_no_diminution_of_rights__safeguards_and_equality_of_opportunity_in_Northern_Ireland.pdf.

[bjos13207-bib-0062] Usherwood, S. , and K. A. M. Wright . 2017. “Sticks and Stones: Comparing Twitter Campaigning Strategies in the European Union Referendum.” British Journal of Politics & International Relations 19, no. 2: 371–388. 10.1177/1369148117700659.

[bjos13207-bib-0063] Walter, B. 2017. “Shamrocks Growing Out of Their Mouths’: Language and the Racialisation of the Irish in Britain.” In Language, Labour and Migration, edited by A. J. Kershen , 57–73. Routledge.

[bjos13207-bib-0064] Women’s Aid Federation for Northern Ireland . 2020. “Covid 19: A Year in Unprecedented Times.” Annual Report 2020‐21. https://www.womensaidni.org/assets/uploads/2021/12/WAFNI‐Annual‐Report‐20‐21.pdf.

[bjos13207-bib-0065] Wright, K. A. M. , R. McAreavey , and R. Donaldson . 2024a. “The Impact of Brexit on Women, Peace and Security in Northern Ireland: Spotlight on Violence Against Women.” Journal of Communication and Media Studies: Journal of Common Market Studies 62, no. 5: 1408–1416. 10.1111/jcms.13663.

[bjos13207-bib-0066] Wright, K. A. M. , R. McAreavey , and R. Donaldson . 2024b. The Impact of Brexit on Women in Northern Ireland. Equality Commission.

[bjos13207-bib-0067] Zambelli, E. , M. Benson , and N. Sigona . 2024. “Brexit Rebordering, Sticky Relationships and the Production of Mixed‐Status Families.” Sociology 58, no. 3: 605–622. 10.1177/00380385231194966.

